# Correlation of quantitative dynamic contrast‐enhanced MRI with microvascular density in necrotic, partial necrotic, and viable liver tumors in a rabbit model

**DOI:** 10.1120/jacmp.v17i5.6314

**Published:** 2016-09-08

**Authors:** Jungwon Moon, Jae‐Hun Kim, Dongil Choi, Jehoon Yang, Min Woo Lee, Yoon‐La Choi, Hyunchul Rhim, MD

**Affiliations:** ^1^ Department of Radiology Samsung Medical Center, Sungkyunkwan University School of Medicine Seoul; ^2^ Laboratory Animal Research Center, Samsung Biomedical Research Institute Seoul; ^3^ Department of Pathology Samsung Medical Center, Sungkyunkwan University School of Medicine Seoul; ^4^ Department of Radiology Kangbuk Samsung Hospital, Sungkyunkwan University School of Medicine Seoul Republic of Korea

**Keywords:** dynamic contrast‐enhanced MRI, pharmacokinetic parameters, microvessel density, VX2 tumor, image biomarker

## Abstract

The purpose of this study was to examine the correlation of quantitative dynamic contrast‐enhanced (DCE) magnetic resonance imaging (MRI) with microvessel density (MVD) in necrotic, partial necrotic, and viable tumors using a rabbit VX2 liver tumor model. Nine rabbits were used for this study. The complete necrotic area (CNA), partial necrotic area (PNA), and viable tumor area (VTA) of liver tumors were experimentally induced by radiofrequency ablation (RFA). DCE‐MRI data were processed based on the extended Kety model to estimate $K^{\text{trans}},v_{e}$ and v_p_ parameters. The boundaries among CNA, PNA, and VTA were delineated based on H&E stain images, and MVD was assessed for each subregion of each VX2 tumor based. There were no correlations between ph‐parameters ($K^{\text{trans}},v_{e}$, and $v_{p}$) and MVD for CNA. For PNA, the $K^{\text{trans}}$ values were positively correlated with the MVD (r=0.8124,p<0.0001). For VTA, we found a positive correlation between $K^{\text{trans}}$ values and the MVD (r=0.5743,p<0.05). Measuring from both the PNA and the VTA, mean $K^{\text{trans}}$ values were positively correlated with mean MVD (r=0.8470,p<0.0001). In a rabbit VX2 liver tumor model, $K^{\text{trans}}$ values correlated well with MVD counts of PNA and VTA in liver tumors.

PACS number(s): 87.19.If MRI

## I. INTRODUCTION

Dynamic contrast‐enhanced (DCE) magnetic resonance imaging (MRI) can provide functional information about tumors such as vascular endothelial proliferation, vascular density, and presence of angiogenesis.^1^, ^2^ DCE‐MRI has recently been used to monitor therapeutic responses to new treatment methods and to evaluate the effects of new antiangiogenic drugs in preclinical and clinical studies.^3^, ^4^, ^5^, ^6^, ^7^

With respect to liver cancer, previous studies have demonstrated the feasibility of using DCE‐MRI as a tool to monitor therapeutic response to antiangiogenic drugs via quantitative DCE‐MRI measurements^(7)^ and semiquantitative DCE‐MRI measurements (i.e., maximal enhancement and peak enhancement).^(8)^ Another study showed the possibility of using semiquantitative DCE‐MRI measurements to identify biomarkers for early prediction of hepatocellular carcinoma (HCC) response after radiotherapy (i.e., slope and peak enhancement).^(9)^ These studies in liver tumors incorporated the basic assumption that the parameters computed from DCE‐MRI data are noninvasive measurements of tumor microenvironments such as microvessel density (MVD). However, no previous studies have investigated correlations between the perfusion parameters and microvessel density in liver cancer.

Previous studies from other cancers have shown conflicting results.^10^, ^11^, ^12^, ^13^ In rectal cancer, Atkin et al.^(10)^ showed a negative correlation between $K^{\text{trans}}$ values and MVD counts. In contrast, Zhang et al.^(11)^ showed a negative correlation between time‐to‐peak enhancement of DCE‐MRI data and MVD counts. In prostate cancer, Schlemmer et al.^(12)^ found a correlation between $k_{\text{ep}}$ ($=K^{\text{trans}}/v_{e}$) values and MVD counts, whereas van Niekerk et al.^(13)^ found no relationship between DCE‐MRI perfusion parameters ($K^{\text{trans}},v_{e}$, and $k_{\text{ep}}$) and MVD counts, but observed positive correlations of $k_{\text{ep}}$ values with MVD counts when tumor‐normal ratio values were used for correcting intersubject variability.

To examine the relationship between perfusion parameters and microvascular density in liver cancer, we quantified DEC‐MRI data based on pharmacokinetic modeling methods using individual arterial input function (AIF) rather than population‐averaged or model AIF, to reflect intersubject variability among parameters, such as decline in cardiac output and increase in atherosclerotic vascular changes. And considering the heterogeneity of tumors in terms of enhancement patterns in DCE‐MRI data, we experimentally induced complete necrotic area (CNA), partial necrotic area (PNA), and viable tumor area (VTA) by performing radiofrequency ablation (RFA) on a rabbit VX2 liver tumor model, and examined the correlation between pharmacokinetic parameters and MVD counts for each subregion of each VX2 liver tumor.

## II. MATERIALS AND METHODS

### A. Rabbit VX2 liver tumor model

All animal work was performed under a license issued by our Institutional Animal Care and Use Committee. A total of nine female New Zealand white rabbits, each weighing between 2.5 and 3.6 kg (mean, 2.7 kg), were used in this study. Before all procedures, rabbits were sedated with intramuscular injections of 1.0 mg/kg acepromazine maleate (Fermenta Animal Health, Kansas City, MO) and 50 mg/kg of ketamine hydrochloride (Ketaject; Phoenix Scientific, St. Joseph, MO). For the rabbit VX2 liver tumor model, VX2 carcinomas were prepared as described previously.^(14)^ Briefly, a midline laparotomy was first performed in which 1–2 VX2 carcinoma fragments approximately 1 mm^3^ in volume were injected into each lobe of the exposed liver using a 16‐gauge needle. Electrocoagulation was then performed at the implantation surface to prevent tumor spillage. A total of 34 VX2 carcinomas were implanted into the livers of nine rabbits. Fifteen of 34 VX2 tumors were used, that were greater than 5 mm in size. We performed percutaneous RFA 14 days after VX2 liver tumor surgery.

### B. Radiofrequency ablation

To prepare for RFA experiments, the epigastrium and back of each rabbit were shaved and sterilized. A wire‐mesh ground pad (10×15 cm) and conductive gel were then placed on the back of the rabbit. Next, a 0.5 cm incision was made for insertion of the RFA needle. The Cool‐tip RFA System (Valleylab; COVIDIEN, Mansfield, MA) with a 1 cm active tip was placed in the presumed target area of the liver under ultrasonography guidance with a 4V1 Acuson probe (Siemens Medical Solutions USA, Malvern, PA). RFA was performed over a period of 3–10 s using a 500‐kHz RF generator (series CC‐3; Radionics, Burlington, MA). To create partial necrotic areas within single tumors, incomplete ablation was performed for the partial volume of certain tumors visualized by ultrasonography. The power output was set to 50W and the applied current and power output of the generator was continuously monitored and recorded during RFA.

### C. MR imaging

MR experiments were performed four days after RFA. All MR images were acquired using a 3.0 T MRI scanner (Intera Achieva 3T, Philips Medical Systems, Best, The Netherlands) with an extremity coil. Before MR experiments, rabbits were sedated and intravenous access was acquired via a marginal ear vein for injection of contrast agent. High spatial resolution coronal T2‐weighted MR images were acquired using a turbo spin echo (TSE) sequence (TE/TR=80/3300 ms,turbo factor=10,FOV=120mm×120mm,matrix=512×512,slice thickness=2mm, and acquisition time=5−6min). DCE‐MR images including whole liver regions were acquired in the coronal plane with a T1‐weighted turbo field echo (TFE) sequence ($\text{TE}/\text{TR}=4.5/2.3\;\text{ms},\text{turbo factor}=30,\text{FOV}=120\text{mm}\times 120\text{mm},\text{matrix}=160\times 160,\text{slice thickness}=2\text{mm},\text{flip angle}=12^{\circ }$, and sampling interval=2.4s). Baseline images were acquired for 5 s, followed by automatic injection of 0.2 ml/kg Gd‐DOTA (Dotarem; Guerbet, France) at 2 mL/s and then a 5 mL normal saline flush with additional acquisition over a total time of 4.8 min (total 120 dynamic images: 2 baseline images, and 118 postcontrast images). For T1 mapping, four precontrast images were acquired with the same imaging parameters using different flip angles (2°, 5°, 10°, and 12°).

### D. DCE‐MRI processing

MR signals did not exhibit a linear relationship with concentration of the contrast agent, and thus MR signal versus time curves were converted into concentration of contrast agent versus time curves. Specifically, the concentration of contrast agent in a given tissue voxel was estimated by determining the difference in longitudinal relaxation rate as follows:

(1) $$C(t)=\left[\frac{1}{T{\left(t\right)}_{1}}-\frac{1}{T_{10}(t)}\right]/r_{1}$$

where $T_{1}(t)$ and $T_{10}$ are the post‐ and precontrast T1 values, respectively, and $r_{1}$ denotes the longitudinal relaxivity ($4.39s^{-1}{\text{mM}}^{-1}$ for blood).^(15)^ The $T_{10}$ value of each voxel was estimated using a variable flip angle method (2°, 5°, 10°, and 12°).^(16)^ After estimating $S_{0}$ and $T_{1}$ values for precontrast images, the postcontrast $T_{1}$ value was obtained for the postcontrast image with 12° flip angles based on the fact that $T_{1}(t)$ can be estimated as a function of time from the SI(t).

For the quantification of DCE‐MR data, we computed pharmacokinetic parameters by nonlinear fitting of concentration time curves into the extended Kety two‐compartment model for each voxel:

(2) $$C_{t}(t)=v_{p}C_{p}(t–t_{0})+K^{\text{trans}}C_{p}(t–t_{0})⊗e^{\left(–\frac{K^{\text{trans}}}{v_{e}}t\right)}$$

where $C_{t}$ is the concentration of contrast agent at the observed tissue, $C_{p}$ is the concentration in blood plasma, $t_{0}$ is the time delay between $C_{p}$ and $C_{t},K^{\text{trans}}$ is the volume transfer constant, $v_{p}$ is the fractional blood plasma volume per unit volume of tissue, and $v_{e}$ is the fractional extravascular extracellular space (EES) per unit volume of tissue. The AIF was manually measured by visual inspection of concentration time curves near the heart in blood vessels $C_{b}$ through the hematocrit for each rabbit:

(3) $$C_{p}(t)=\frac{C_{b}(t)}{\left(1–H_{ct}\right)}$$

where the $H_{\text{ct}}$ is the hematocrit, estimated in rabbits to be 0.45.^(17)^ For estimation of $K^{\text{trans}},v_{p},v_{e}$, and $t_{0}$, nonlinear least squares fitting was implemented using the lsqnonlin function from the MATLAB Optimization Toolbox (MathWorks Inc., Natick, MA).

Region of interest (ROI) was manually defined on high‐resolution T2‐weighted images by one radiologist, who also took into account the pathologic findings of overall tumor extent. Each manually defined ROI was resampled into a low‐resolution image (DCE‐MR image space) and used for quantitative analysis of DCE‐MR images. For each ROI, we computed $K^{\text{trans}},v_{p}$, and $v_{e}$ parameters from DCE‐MRI data in voxel‐by‐voxel manner. The ph‐parameters were averaged across 2nd neighborhood voxels (9 voxels) for each CNA, PNA, and VTA regions based on the $K^{\text{trans}}$ parametric map.

### E. Pathologic analysis

After MR experiments, rabbits were euthanized by intravenous bolus injection of potassium chloride. Livers were subsequently removed and sectioned in the coronal plane similar to that used for MR imaging. Tumor specimens were divided into anterior and posterior sections. One specimen of each pair was fixed in 10% neutral buffered formalin, embedded in paraffin and then sliced into 4 μm sections for hematoxylin and eosin (H&E) staining and CD31 immunohistochemistry analysis.

For immunohistochemical staining with anti‐CD31 antibody, paraffin‐embedded sections were deparaffinized in xylene, rehydrated in graded alcohol, and transferred to 0.01 M phosphate‐buffered saline (pH 7.4). After heat induced epitope retrieval with citrate buffer (pH 6.0; Dako, Carpinteria, CA) for 3 min at 121°C to reveal hidden antigen epitopes, endogenous peroxidase was blocked with 3% hydrogen peroxide in PBS for 10 min at room temperature. After washing in PBS buffer, sections were treated with serum‐free protein blocking solution (Dako) for 20 min at room temperature to block nonspecific binding. Subsequently, sections were incubated with anti‐CD31 rabbit polyclonal antibody (1/200; Novus Biologicals, Littleton, CO) for 60 min at room temperature. After washing in PBS, the sections were incubated for 30 min at room temperature with HRP‐labeled polymer conjugated secondary antibodies against rabbit IgG (Dako). The color reaction was developed using the ready‐to‐use DAB (3,3′‐diaminobenzidine) substrate‐chromogen solution (Dako) for 5 min and then washed with distilled water. Finally, sections were lightly counterstained with Mayer's hematoxylin for 1 min. All sections were also stained with H&E using standard techniques for histological analysis.

### F. Microvessel density analysis

In order to examine the relationships between pharmacokinetic parameters estimated from the DCE‐MR data and MVD estimated from the histopathological data, an experienced pathologist delineated boundaries among the CNA, the PNA, and the VTA on H&E stain images. We defined the CNA as the regions composed of only necrotic tissues, the PNA as the transition zone from necrotic tissues to viable tumors, and the VTA as regions composed only of viable tumors. Segmented histopathologic images were used as reference images to assess microvessel density.

For MVD analysis, each side was imaged at low magnification (×12.5) to identify CNA, PNA and VTA. For each subregion, five areas were randomly selected. These regions were then imaged at high magnification (×200) and individual microvessels were counted. Any clearly brown stained endothelial cells were counted as individual vessels. The average value of the five areas was used as the representative value of MVD for each CNA, PNA, and VTA.

### G. Statistical analysis

For statistical analysis, we used Wilcoxon signed‐rank test to examine differences in pharmacokinetic parameters, and differences of MVD between CNA, PNA, and VTA. Pearson correlation analysis was used to examine the relationships between pharmacokinetic parameters and MVD counts in each CNA, PNA, and VTA. P‐values of 0.05 or less were considered statistically significant.

## III. RESULTS

### A. VX2 tumors after RFA

Two rabbits died before MR experiments. We excluded VX2 tumors with nonenhanced patterns in DCE‐MRI data or that were smaller than 5 mm in size. Fifteen of the total experimentally induced 34 VX2 tumors were included, and the mean size of 15 VX2 tumors was 14 mm, with a range of 8 mm to 22 mm.

### B. MR images and histopathologic images of VX2 tumors

**Figure 1:**
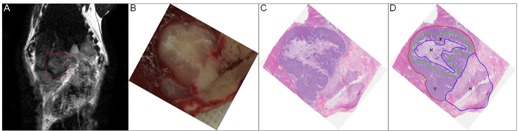
T2‐weighted MR image (a) of a rabbit VX2 liver tumor model after RFA; (b) histopathologic specimens corresponding to the VX2 tumor shown in (a); (c) H&E stain; (d) the complete necrotic tissue areas, partial necrotic areas, and viable tumor areas were manually drawn on H&E stain images. N: complete necrosis (blue boundary), P: partial necrosis (green boundary), V: viable area (red boundary)

Figure 1 shows a representative VX2 tumor on T2‐weighted MR (Fig. 1(a)), the corresponding histopathologic specimen (Fig. 1(b)), and an H&E stain image (Fig. 1(c)). We did not detect heterogeneity of VX2 tumors on T2‐weighted MR images, but the H&E stain images clearly showed heterogeneous VX2 tumors after RFA, composed of completely necrotic tissues and viable tumors. Based on the H&E stain images, boundaries were delineated among the CNA, PNA, and VTA for microvessel density analysis (Fig. 1(d)).

### C. Pharmacokinetic parameters from DCE‐MRI data

**Figure 2:**
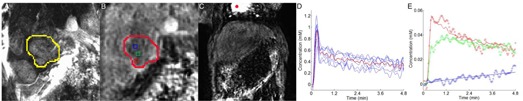
Quantification of DCE‐MRI data: (a) tumor boundary manually drawn on T2‐weighted MR image for quantification of DCE‐MRI data (yellow boundary); (b) the manually drawn ROIs were resampled to delineate tumor boundaries on the DCE‐MRI data (red boundary); (c) the manually defined AIF on the DCE‐MRI data at 12 s (red circle). The arterial input function (d) for pharmacokinetic modeling DCE‐MRI data; the solid red line represents the mean concentration time curve of AIFs for 7 rabbits. The nonlinear curve fitting (e) of the observed concentration time curves. In (e), the circles represent the observed concentration time curve in the complete necrotic area (blue circle), partial necrotic areas (green circle), and viable tumor area (red circle). The solid line represents fitted curves. The observed concentration time curve in the complete necrotic tissue areas was extracted from the blue box, in the partial necrotic areas from the green box, and in the viable tumor areas from the red box, as shown in (b).

**Figure 3:**
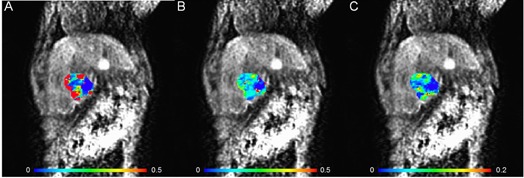
Pharmacokinetic parametric maps for $K^{\text{trans}}$ (a), $v_{e}$ (b), and vp (c). Blue represents 0, and red represents max value in the color bar.

For the quantification of DCE‐MRI data, we used the extended Kety two‐compartment model to estimate $K^{\text{trans}},v_{e}$ and $v_{p}$ parameters. The tumor boundaries were manually delineated on T2‐weighted MR images as shown in Fig 2(a). The manually defined ROIs on T2‐weighted MR images were resampled into the DCE‐MRI data for DCE‐MRI processing, as shown in Fig. 2(b). Figure 2(c) shows an example of the manually defined AIF near the heart, and Fig. 2(d) shows the AIFs for each rabbit. VX2 tumors exhibited heterogeneous properties in concentration profiles. Figure 2(e) shows representative concentration profiles in the CNA, PNA, and VTA. The concentration versus time curves in the VTA showed high wash‐in and wash‐out enhancement patterns ($K^{\text{trans}}=0.4488$; $v_{e}=0.0840,v_{p}=0.0237$), whereas the concentration versus time curves in the CNA showed slow or nonenhancement patterns ($K^{\text{trans}}=0.0039$; $v_{e}=0.1870,v_{p}=0.009$). The concentration versus time curves in the PNA showed enhancement patterns between those of the viable tumor areas and the complete necrotic tissues

($K^{\text{trans}}=0.0873$; $v_{e}=0.0440,v_{p}=0.0113$). Figure 3 shows pharmacokinetic parametric maps for $K^{\text{trans}},v_{e}$, and $v_{p}$. Statistical analysis revealed that there were significant differences in $K^{\text{trans}}$ value between the CNA and the PNA (p<0.001), between the PNA and the VTA (p<0.001), and between the CNA and the VTA (p<0.001). We observed significant differences in the $v_{p}$ parameter between the CNA and the PNA (p<0.05), and between the CNA and the VTA (p<0.05), but not between the PNA and the VTA. We did not observe significant differences of $v_{e}$ between the CNA, the PNA, and the VTA. Table 1 shows a summary of the $K^{\text{trans}},v_{e}$, and $v_{p}$ parameters for each tumor.

**Table 1:** Descriptive statistics for pharmacokinetic parameters from DCE‐MRI data and for MVD counts from CD31 staining in VX2 tumors.

	*CNA*				*PNA*				*VTA*			
*Tumor*	$K^{\text{trans}}$	$v_{e}$	$v_{p}$	*MVD*	$K^{\text{trans}}$	$v_{e}$	$v_{p}$	*MVD*	$K^{\text{trans}}$	$v_{e}$	$v_{p}$	*MVD*
1	0.00	0.33	0.00	0.00	0.03	0.03	0.01	4.60	0.03	0.04	0.02	15.40
2	0.04	0.04	0.03	1.80	0.11	0.07	0.03	6.40	0.48	0.08	0.02	17.20
3	0.01	0.17	0.01	0.00	0.02	0.05	0.02	3.40	0.03	0.05	0.03	16.60
4	0.00	0.20	0.01	0.40	0.09	0.04	0.01	6.60	0.45	0.08	0.02	16.40
5	0.05	0.07	0.08	0.60	0.34	0.16	0.08	5.25	1.13	0.22	0.09	14.40
6	0.03	0.24	0.03	1.00	0.16	0.25	0.02	7.60	1.00	0.17	0.08	12.40
7	0.02	0.05	0.01	0.20	0.23	0.29	0.13	5.60	0.60	0.26	0.03	16.80
8	0.02	0.66	0.00	0.40	0.16	0.16	0.02	5.00	0.71	0.17	0.02	14.67
9	0.28	0.14	0.01	1.00	1.50	0.19	0.02	14.60	2.47	0.19	0.03	31.40
10	0.07	0.22	0.00	0.60	0.17	0.21	0.06	7.00	1.17	0.23	0.06	26.00
11	0.02	0.56	0.01	3.40	0.23	0.13	0.04	4.40	1.07	0.24	0.06	8.20
12	0.05	0.10	0.01	1.40	0.16	0.08	0.02	4.25	0.42	0.08	0.02	11.00
13	0.04	0.04	0.02	1.20	0.91	0.21	0.00	21.00	4.03	0.13	0.00	23.40
14	0.01	0.59	0.00	0.20	0.16	0.08	0.03	5.00	0.85	0.11	0.01	18.40
15	0.02	0.05	0.01	4.50	0.13	0.06	0.02	5.00	0.56	0.07	0.00	11.00

CNA=complete necrotic area; PNA=partial necrotic area; VTA=viable tumor area.

### D. Microvessel density of subregions for VX2 tumors

**Figure 4:**
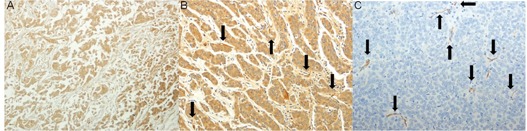
Representative image immunohistochemically stained with anti‐CD31 antibody for complete necrotic area (a), partial necrotic area (b), and viable tumor area (c). The black arrows indicate the CD31‐postive vessels (shown in brown, magnification x200).

To examine the relationship between pharmacokinetic parameters and microvessel density, we manually defined boundaries among the CNA, the PNA, and the VTA based on H&E stain images for each VX2 tumor. MVD was counted for each subregion of a VX2 tumor (Fig. 4). We observed significant differences in MVD count between the CNA and the PNA (p<0.001), between the CNA and the VTA (p<0.001), and between the PNA and the VTA (p<0.001). Table 1 summarizes microvessel density for CNA, PNA, and VTA for each VX2 tumor.

### E. Correlations between ph‐parameters and MVD for subregions

**Figure 5:**
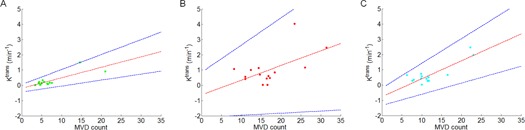
Scatter plots of $K^{\text{trans}}$ and MVD counts in partial necrotic areas (a), viable tumor areas (b), and in both partial necrotic areas and viable tumor areas (c). The red dotted line represents the coefficient estimates. The blue dotted line represents 95% confidence intervals for the coefficient estimates.

As assessed using CNA, the ph‐parameters were not significantly correlated with MVD count for $K^{\text{trans}}$ (r=0.0075,p=0.9788), $v_{e}$ (r=−0.1153,p=0.6824), or $v_{p}$ (r=0.0245,p=0.9309). For PNA, the $K^{\text{trans}}$ values were positively correlated with the MVD count (r=0.8124,p<0.001, Fig. 5(a)), but not with $v_{e}$ (r=0.4228,p=0.1164) or $v_{p}$ (r=−0.2750,p=0.3212). For VTA, we also observed a positive correlation between the $K^{\text{trans}}$ values and the MVD count (r=0.5743,p<0.05, Fig. 5B), but not for $v_{e}$ (r=0.1743,p=0.5345) or $v_{p}$ (r=−0.1340,p=0.6340). Measuring from both the PNA and the VTA, the mean $K^{\text{trans}}$ values were positively correlated with the mean MVD count (r=0.8470,p<0.0001, Fig. 5(c)), but not $v_{e}$ (r=0.3043,p=0.2701) or $v_{p}$ (r=−0.1943,p=0.4877). In correlation analyses, we found positive correlations between the $K^{\text{trans}}$ values and the MVD count in the PNA and the VTA, but not in the

CNA. When measuring the mean $K^{\text{trans}}$ values and the mean MVD count from the PNA and the VTA, the correlation coefficient was higher than when measuring from the PNA or the VTA.

## IV. DISCUSSION

In this study, we examine the relationship between pharmacokinetic parameters and MVD in a rabbit VX2 liver tumor model, and demonstrate significant positive correlations of $K^{\text{trans}}$ values and MVD counts in the PNA and the VTA.

$K^{\text{trans}}$ is the volume transfer constant between the intravascular ($v_{p}$) and extravascular extracellular compartment ($v_{e}$). This exchange rate between $v_{p}$ and $v_{e}$ spaces depends on vascular perfusion factors such as blood flow and permeability surface area product. Thus, $K^{\text{trans}}$ may be interpreted as tumor blood flow or vascular permeability, depending on whether the underlying tumor condition is flow‐limited or permeability‐limited.^(18)^ In this study, the increased numbers of blood vessels counted from CD31 stain images indicate increased blood flow of contrast agent or increased permeability surface area in the PNA and the VTA of VX2 tumors, reflecting increased $K^{\text{trans}}$ value in DCE‐MRI data, in turn showing positive correlations between MVD and $K^{\text{trans}}$ values.

We observed strong correlations when measuring both the PNA and the VTA rather than the PNA or the VTA alone. Interestingly, the correlation was stronger when measured from the PNA than the VTA. The ph‐parameters of DCE‐MRI data and the MVD counts of CD31 stained data are strongly affected by the definition of the tumor ROI.^(19)^ Inclusion of voxels in the CNA artificially lowered the mean of the values, whereas inclusion of the voxels in VTA artificially increased the mean of the values while ignoring tumor heterogeneity. As shown in Table 1, we found that intersubject variability is higher in VTA than PNA for both ph‐parameters and MVD counts. This large intersubject variability may explain the lower correlation in VTA compared with that in PNA. For robust correlations, our findings suggest that ph‐parameters and the MVD counts should be measured from both the PNA and the VTA to reflect tumor heterogeneity, while excluding the CNA.

There may have been possible errors in the individual AIF measurement such as inflow effects and inaccurate $T_{1}$ measurement caused by $B_{1}$ field inhomogeneity. There may be also a possibility of the underestimation of the individual AIF due to the partial volume effects. In this study, to minimize the partial volume effects, we carefully defined the AIF voxels near heart by visual inspection of the shape of concentration time curves (i.e., high peak, narrow shape, and quick wash‐in and wash‐out).

DCE‐MRI data obtained from the liver during free breathing leads to artifacts in pharmacokinetic parameters, particularly in areas close to liver boundaries (such as the diaphragm). Conventional registration methods are likely to fail with DCE‐MRI data because of important local intensity changes across different time‐points. New methods have been proposed using tracer kinetic model‐driven registration,^(20)^ progressive principal component registration,^(21)^ and robust principal component registration,^(22)^ but it is difficult to apply these methods into practical and clinical setting due to its complexity of algorithm. To minimize motion artifacts, in this study, we included only large VX2 tumors (>5mm), which are less affected by motion.

More sophisticated pharmacokinetic models are required for the quantification of DCE‐MRI data in the liver. Considering the dual bloods flows (arterial and venous) into tissues of the liver, the extended Kety model (two compartments with arterial input function) could not perfectly explain the hemodynamic changes of contrast agent in the liver. In our study, we assumed that a single arterial input (extended Kety model) may suffice to approximate the vascular behavior in VX2 tumors, because hypervascular VX2 tumors are predominantly supplied by hepatic arterial vessels. Many previous studies have demonstrated the usefulness of the extended Kety model for studying liver tumors, in which the predominant blood supply comes from the hepatic arterial blood flow.^23^, ^24^, ^25^ Possible correlations of pharmacokinetic parameters based on the two compartments with arterial and venous blood flows with histopathologic microvascular parameters should be the focus of future studies.

## V. CONCLUSION

We demonstrated the strong correlations of $K^{\text{trans}}$ values with MVD counts in the PNA and the VTA in a rabbit VX2 liver tumor model. Thus, our results provide direct evidence that $K^{\text{trans}}$ values reflect microvascular information regarding liver tumors, suggesting the possibility of DCE‐MRI tool to generate image biomarkers for prediction of response to therapeutic treatments such as antiangiogenic drugs, RFA, and radiotherapy in patients with HCC.

## ACKNOWLEDGMENTS

This research was performed with the support of a grant (code A102142 to D. Choi) from the Korean Healthcare Technology R&D Project, Ministry for Health, Welfare & Family Affairs, Republic of Korea. This research was also supported by Basic Science Research Program (to JH Kim) through the National Research Foundation of Korea (NRF) funded by the Ministry of Education, Science and Technology (NRF‐2012R1A1A2003618).

## COPYRIGHT

This work is licensed under a Creative Commons Attribution 3.0 Unported License.

## References

[1] Raatschen HJ , Simon GH , Fu Y , et al. Vascular permeability during antiangiogenesis treatment: MR imaging assay results as biomarker for subsequent tumor growth in rats. Radiology. 2008;247(2):391–99. 18372448 10.1148/radiol.2472070363 PMC4423757

[2] Hillman GG , Singh‐Gupta V , Zhang H , et al. Dynamic contrast‐enhanced magnetic resonance imaging of vascular changes induced by sunitinib in papillary renal cell carcinoma xenograft tumors. Neoplasia. 2009;11(9):910–20. 19724685 10.1593/neo.09618 PMC2735805

[3] Kim JH , Im GH , Yoon J , et al. Dynamic contrast‐enhanced MRI for assessing therapeutic response of choroidal neovascularization in a rat model. Invest Ophthalmol Vis Sci. 2012;53(12):7693–700. 23111615 10.1167/iovs.12-9805

[4] Kim JH , Kim CK , Park BK , Park SY , Huh SJ , Kim B . Dynamic contrast‐enhanced 3‐T MR imaging in cervical cancer before and after concurrent chemoradiotherapy. Eur Radiol. 2012;22(11):2533–39. 22653283 10.1007/s00330-012-2504-4

[5] Kim YS , Lim HK , Kim JH , et al. Dynamic contrast‐enhanced magnetic resonance imaging predicts immediate therapeutic response of magnetic resonance‐guided high‐intensity focused ultrasound ablation of symptomatic uterine fibroids. Invest Radiol. 2011;46(10):639–47. 21654495 10.1097/RLI.0b013e318220785c

[6] Yang J , Kim JH , Im GH , et al. Evaluation of antiangiogenic effects of a new synthetic candidate drug KR‐31831 on xenografted ovarian carcinoma using dynamic contrast enhanced MRI. Korean J Radiol. 2011;12(5):602–10. 21927562 10.3348/kjr.2011.12.5.602 PMC3168802

[7] Song KD , Choi D , Lee JH , et al. Evaluation of tumor microvascular response to brivanib by dynamic contrast‐enhanced 7‐T MRI in an orthotopic xenograft model of hepatocellular carcinoma. AJR Am J Roentgenol. 2014;202(6):W559–66. 24848850 10.2214/AJR.13.11042

[8] Wang J , Chen LT , Tsang YM , Liu TW , Shih TT . Dynamic contrast‐enhanced MRI analysis of perfusion changes in advanced hepatocellular carcinoma treated with an antiangiogenic agent: a preliminary study. AJR Am J Roentgenol. 2004;183(3):713–19. 15333360 10.2214/ajr.183.3.1830713

[9] Liang PC , Ch'ang HJ , Hsu C , Tseng SS , Shih TT , Wu Liu T . Dynamic MRI signals in the second week of radiotherapy relate to treatment outcomes of hepatocellular carcinoma: a preliminary result. Liver Int. 2007;27(4):516–28. 17403192 10.1111/j.1478-3231.2007.01456.x

[10] Atkin G , Taylor NJ , Daley FM , et al. Dynamic contrast‐enhanced magnetic resonance imaging is a poor measure of rectal cancer angiogenesis. Br J Surg. 2006;93(8):992–1000. 16673354 10.1002/bjs.5352

[11] Zhang XM , Yu D , Zhang HL , et al. 3D dynamic contrast‐enhanced MRI of rectal carcinoma at 3T: correlation with microvascular density and vascular endothelial growth factor markers of tumor angiogenesis. J Magn Reson Imaging. 2008;27(6):1309–16. 18504761 10.1002/jmri.21378

[12] Schlemmer HP , Merkle J , Grobholz R , et al. Can pre‐operative contrast‐enhanced dynamic MR imaging for prostate cancer predict microvessel density in prostatectomy specimens? Eur Radiol. 2004;14(2):309–17. 14531000 10.1007/s00330-003-2025-2

[13] van Niekerk CG , van der Laak JA , Hambrock T , et al. Correlation between dynamic contrast‐enhanced MRI and quantitative histopathologic microvascular parameters in organ‐confined prostate cancer. Eur Radiol. 2014;24(10):2597–605. 25033819 10.1007/s00330-014-3301-z

[14] Choi BI , Lee DH , Han MC . Necrotic areas in VX2 carcinoma of rabbits. Correlation of magnetic resonance imaging and pathologic appearance. Invest Radiol. 1993;28(1):33–38. 8425850 10.1097/00004424-199301000-00010

[15] Brix G , Kiessling F , Lucht R , et al. Microcirculation and microvasculature in breast tumors: pharmacokinetic analysis of dynamic MR image series. Magn Reson Med. 2004;52(2):420–29. 15282828 10.1002/mrm.20161

[16] Deoni SC , Rutt BK , Peters TM . Rapid combined T1 and T2 mapping using gradient recalled acquisition in the steady state. Magn Reson Med. 2003;49(3):515–26. 12594755 10.1002/mrm.10407

[17] Chen B , Zhang Y , Song X , Wang X , Zhang J , Fang J . Quantitative estimation of renal function with dynamic contrast‐enhanced MRI using a modified two‐compartment model. PLoS One. 2014;9(8):e105087. 25141138 10.1371/journal.pone.0105087 PMC4139329

[18] Tofts PS , Brix G , Buckley DL , et al. Estimating kinetic parameters from dynamic contrast‐enhanced T(1)‐weighted MRI of a diffusable tracer: standardized quantities and symbols. J Magn Reson Imaging. 1999;10(3):223–32. 10508281 10.1002/(sici)1522-2586(199909)10:3<223::aid-jmri2>3.0.co;2-s

[19] Jackson A , O'Connor JP , Parker GJ , Jayson GC . Imaging tumor vascular heterogeneity and angiogenesis using dynamic contrast‐enhanced magnetic resonance imaging. Clin Cancer Res. 2007;13(12):3449–59. 17575207 10.1158/1078-0432.CCR-07-0238

[20] Buonaccorsi GA , O'Connor JP , Caunce A , et al. Tracer kinetic model‐driven registration for dynamic contrast‐enhanced MRI time‐series data. Magn Reson Med. 2007;58(5):1010–19. 17969122 10.1002/mrm.21405

[21] Melbourne A , Atkinson D , White MJ , Collins D , Leach M , Hawkes D . Registration of dynamic contrast‐enhanced MRI using a progressive principal component registration (PPCR). Phys Med Biol. 2007;52(17):5147–56. 17762077 10.1088/0031-9155/52/17/003

[22] Hamy V , Dikaios N , Punwani S , et al. Respiratory motion correction in dynamic MRI using robust data decomposition registration — application to DCE‐MRI. Med Image Anal. 2014;18(2):301–13. 24322575 10.1016/j.media.2013.10.016

[23] Hsu CY , Shen YC , Yu CW , et al. Dynamic contrast‐enhanced magnetic resonance imaging biomarkers predict survival and response in hepatocellular carcinoma patients treated with sorafenib and metronomic tegafur/uracil. J Hepatol. 2011;55(4):858–65. 21338641 10.1016/j.jhep.2011.01.032

[24] Esposito A , Palmisano A , Maffi P , et al. Liver perfusion changes occurring during pancreatic islet engraftment: a dynamic contrast‐enhanced magnetic resonance study. Am J Transplant. 2014;14(1):202–29. 24219129 10.1111/ajt.12501

[25] Hirashima Y , Yamada Y , Tateishi U , et al. Pharmacokinetic parameters from 3‐Tesla DCE‐MRI as surrogate biomarkers of antitumor effects of bevacizumab plus FOLFIRI in colorectal cancer with liver metastasis. Int J Cancer. 2012;130(10):2359–65. 21780098 10.1002/ijc.26282

